# An instrumental variable analysis of body mass index and risk of long-term sick leave: the HUNT Study, Norway

**DOI:** 10.1007/s10654-025-01299-6

**Published:** 2025-09-04

**Authors:** Karoline Moe, Eivind Schjelderup Skarpsno, Tom Ivar Lund Nilsen, Silje L. Kaspersen, Solveig Osborg Ose, David Carslake, Paul Jarle Mork, Lene Aasdahl

**Affiliations:** 1 Department of Public Health and Nursing, Faculty of Medicine and Health Sciences, Norwegian University of Science and Technology, Postboks 8905, 7491 Trondheim, Norway; 2 Department of Neurology and Clinical Neurophysiology, St. Olavs Hospital, Trondheim, Norway; 3 Clinic of Emergency Medicine and Prehospital Care, St. Olav’s Hospital, Trondheim University Hospital, Trondheim, Norway; 4 Department of Health Research, SINTEF Digital, Trondheim, Norway; 5 Medical Research Council (MRC) Integrative Epidemiology Unit at the University of Bristol, Bristol, UK; 6 Population Health Sciences, Bristol Medical School, Faculty of Health Sciences, University of Bristol, Bristol, UK; 7 Unicare Helsefort Rehabilitation Centre, Rissa, Norway

**Keywords:** Instrumental variable, Offspring as instrument, Sick leave, Body mass index

## Abstract

A more comprehensive understanding of the causal relationships between body mass index (BMI) and sick leave is needed**.** We aimed to examine the effect of BMI on the risk of cause-specific and all-cause long-term sick leave using an instrumental variable approach. The study included 21,918 adults participating in the two latest surveys of the population-based HUNT Study (HUNT3, 2006–2008 and HUNT4, 2017–2019) linked with registry data on cause-specific sick leave, including musculoskeletal and mental disorders. We used Cox regression to estimate risk of long-term sick leave per standard deviation (SD) increase in z-score of BMI, applying both conventional analysis of own BMI and instrumental variable analysis based on offspring BMI. In the conventional analyses, hazard ratios per SD increase in z-score of BMI ranged from 1.04 (95% confidence interval (CI) 0.99–1.08) for mental health disorders in women to 1.17 (95% CI 1.13–1.22) for musculoskeletal disorders in men. The instrumental variable approach supported that higher BMI increased the risk of long-term sick leave, except for sick leave due to mental health disorders in men. The analyses suggested that offspring BMI as an instrument is not independent of shared confounding. The results from both the conventional and instrumental variable analyses show that higher BMI increases the risk of long-term sick leave, except for sick leave due to mental health disorders in men. The instrumental variable method is likely to remove bias due to reverse causation, but residual bias due to shared confounding factors cannot be ruled out.

**Supplementary Information:**

The online version contains supplementary material available at 10.1007/s10654-025-01299-6.

## Introduction

To maintain or improve work ability and prevent sick leave, more knowledge is needed on the causal relationships between relevant health exposures and work participation. Higher body mass index (BMI) is associated with sick leave and disability benefits due to musculoskeletal disorders [1, 2] and mental health disorders [3], which are the leading causes of sickness absence statistics [4]. As the prevalence of obesity and population mean BMI are increasing globally [5], leading to increased healthcare costs [6], a deeper understanding of the causal relationships between BMI and work related outcomes is needed. Since conventional observational studies are prone to residual confounding and reverse causation, it is important to triangulate evidence by using causal approaches with diverse sources of bias [7].

Instrumental variable analysis can, under certain assumptions, obtain estimates of the causal effect of exposures that are free from bias due to reverse causation, measurement error and confounding [8]. A valid instrument should be i) associated with the exposure of interest, ii) independent of the outcome, and iii) independent of confounding factors shared with the outcome [9]. One approach is to use offspring exposures as instrumental variables for the same exposures in the parents. This method takes advantage of intergenerational relationships to improve causal inference [10–13]. Although confounding factors that are intergenerationally associated (such as behavioral and socioeconomic factors) could leave remaining bias in the estimates, an instrumental variable approach using offspring as an instrument would still be effective to handle reverse causation caused by ill health. Thus, due to the unknown role of reverse causation on the association between BMI and long-term sickness absence, it would be valuable to use an instrumental variable analysis together with conventional analysis in terms of triangulating evidence [7].

Offspring BMI has been used as an instrumental variable in several studies of mortality [11, 12, 14–16] and healthcare costs [6], but only one previous study has applied the method in the context of employment disability [10]. However, the latter study was based on self-reported exposure and outcome, showing no evidence that body weight causes employment disability [10]. Data from the population-based HUNT Study and the Norwegian Labour and Welfare Administration (NAV), alongside registry data on family relations, offers a unique possibility to study the association between BMI and risk of long-term sick leave using an instrumental variable approach. Therefore, the aim of this study was to examine the effect of BMI on the risk of cause-specific and all-cause long-term sick leave using offspring BMI as an instrument along with conventional analysis.

## Methods

### Study sample

We included participants from the third and fourth consecutive surveys of the population-based HUNT Study, as registry data on sick leave was first available from year 2000. All inhabitants aged 20 years and above in Nord-Trøndelag County, Norway, were invited to participate in HUNT3 (2006–08) and HUNT4 (2017–19). Adolescents aged 13–19 years were invited to participate in Young-HUNT3 (2006–08) and Young-HUNT4 (2017–19). Details of the different HUNT surveys are described elsewhere [17–20].

**Fig. 1:**
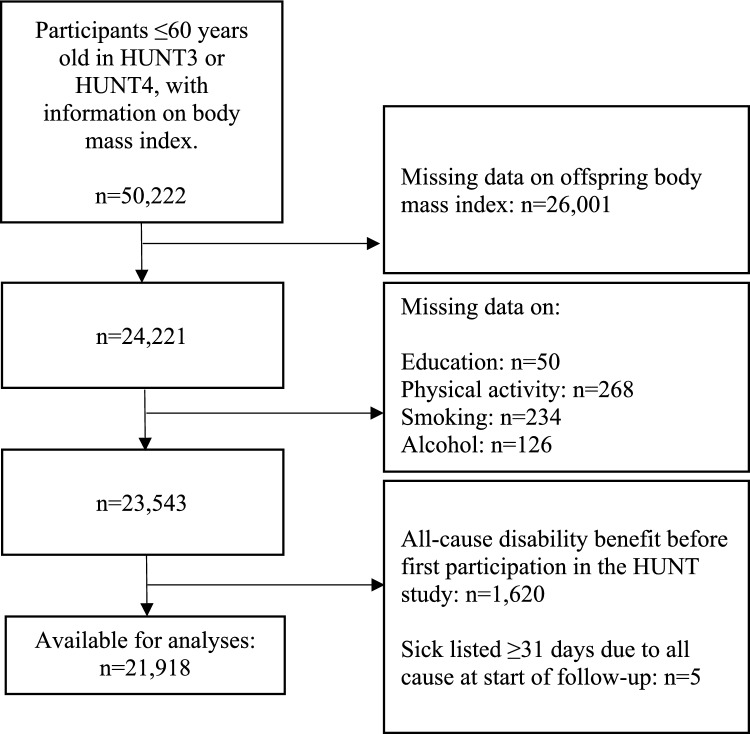
Flowchart. Participants drawn from HUNT3 (2006–08) and HUNT4 (2017–19). Offspring drawn from HUNT3, YoungHUNT3 (2006–08), HUNT4 or YoungHUNT4 (2017–19)

Registry data on family relations were obtained from Statistics Norway, and data from the different sources were linked using the national identity number. For those who participated in more than one survey, we used data from their first participation. Information on offspring BMI were selected from HUNT3, HUNT4, YoungHUNT3 and YoungHUNT4. If a participant had information from several offspring, the oldest offspring from the earliest participation was selected. A total of 50,222 participants ≤ 60 years with information on BMI participated in either HUNT3 or HUNT4 (Fig. 1). The upper age for inclusion was chosen to reduce competing risk events, such as early age retirement. Participants were excluded if they lacked data on offspring BMI, or their own education, smoking, alcohol consumption or physical activity. Participants were also excluded if they received disability benefits or had been sick listed for ≥ 31 days at participation. This left 21,918 participants (9606 men and 12,312 women) with complete information available for statistical analyses (Fig. 1), whereas 14,829 offspring contributed to the instrumental variable analyses.

### Long-term sick leave

We used registry data from NAV to obtain data on medical certified sick leave and disability benefits, including diagnoses. Long-term sick leave was defined as the first period lasting at least 31 consecutive days after the start of follow-up (date of participation in HUNT), and the start date of such a period was used as the time of the outcome. Sick leave due to musculoskeletal disorders was identified based on the 9th and 10th revisions of the International Classification of Diseases (ICD-10 codes M00-M99 and their ICD-9 equivalents) and the International Classification of Primary Care (ICPC1 and ICPC2, codes L00-L99). Sick leave due to mental disorders was identified from the codes F00-F99 (ICD-10 codes and their ICD-9 equivalents) and P00-P99 (ICPC1 and ICPC2). Follow-up lasted until the date of a long-term sick leave, all-cause disability benefit, 65 years, emigration, death, or end of follow-up (31^st^ December 2021), whichever came first.

### Body mass index

Body mass index (BMI, kg/m^2^) was calculated from baseline measurements of weight and height. BMI was then transformed into z-scores specific to age category (< 30, 30–39.9, 40–49.9, ≥ 50 years) at baseline, sex, and HUNT survey. For offspring <18 years at participation, we used age- and sex-specific BMI measures based on underlying Lambda-Mu-Sigma curves from the International Obesity Task Force to obtain z-scores [21].

### Other variables

Data on disability benefits were obtained from NAV and defined as the first day of the month the benefit was registered, regardless of grading. Information on educational attainment was obtained from Statistics Norway using the highest completed education level at the date of HUNT participation. Education was classified as (1) "no completed education", (2) "compulsory school", (3) "upper secondary school", (4) "tertiary vocational education", (5) "higher education, undergraduate level", and (6) "higher education, postgraduate level". Smoking status was categorized as “never smoked”, “ex-smoker”, “occasional smoker”, and “daily smoker”. Alcohol consumption in the last 12 months was categorized as “never drunk alcohol”, “not at all the last year”, “once a month or less”, “2–4 times a month”, “2–3 times a week”, and “4 times or more a week”. Leisure time physical activity per week was self-reported according to usual frequency (“0”, “< 1”, “1”, “2–3”, or “≥ 4 times per week”), intensity (“no sweat/not out of breath”, “sweat/out of breath”, or “taking it all out”) and duration of each session (“< 15”, “15–30”, “31–60”, or “>60 min”). Based on this information, participants were classified into (1) “inactive” (no light or hard physical activity), (2) “low activity” (< 3 h light and no hard activity), (3) “moderate activity” (≥ 3 h light activity and/or < 1 h hard activity), and (4) “high activity” (any light and >1 h hard activity). Occupation was categorized according to the international Erikson Goldthorpe Portocarero (EGP) social class scheme [22]. There are originally 6 categories (class I, II, III, IV, V+VI or VII), where class I is considered to have the highest socioeconomic status [22, 23]. We chose to additionally include a category (VIII) representing those classified as military, unknown occupation, or missing. A more detailed description can be found in the electronic supplementary material.

### Statistical analysis

Participants’ characteristics were summarized within categories of both own and offspring BMI z-score (<− 1, − 1 to 1, or >1 standard deviation [SD]) and also displayed as mean difference or odds ratio per SD increase in z-score from linear or logistic regression, and presented separately for women and men.

Risk of long-term sick leave per SD increase in z-score of own BMI and offspring BMI was estimated separately for women and men and expressed as hazard ratios (HRs) from Cox regressions using age as the time axis. Corresponding incidence rates per 1,000 person-years at risk are also presented. Separate analyses were performed for the different event diagnoses (i.e., sick leave due to musculoskeletal, mental, or all-causes). All models included an interaction term between parental age at baseline as a cubic spline with five knots and wave of HUNT survey, to account for secular trends and cohort effects. The fully adjusted models were in addition adjusted for education, occupation, smoking status, alcohol consumption, and physical activity. In corresponding analyses, we also estimated HRs of all-cause sick leave according to categories of BMI (“< 18.5”, “18.5–19.9”, “20.0–24.9”, “25.0–29.9”, “30.0–34.9”, and “≥ 35.0” kg/m^2^).

**Fig. 2:**
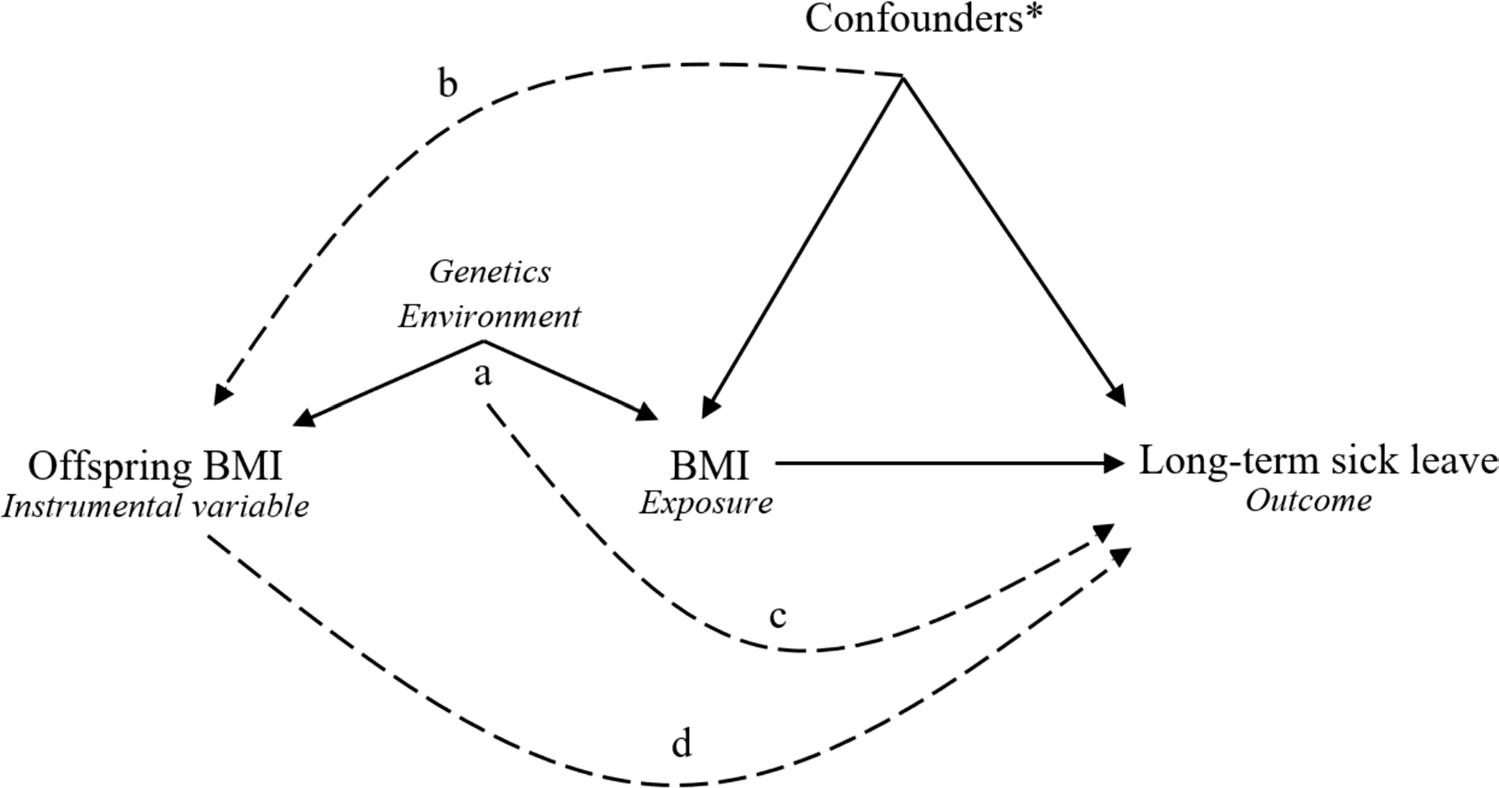
Directed acyclic graph of the possible causal effect of body mass index (BMI) on long-term sick leave. Solid lines represent causal effects, and dotted lines represent potential violations of assumptions. Instrumental variable analysis assumes that the instrument is associated with the exposure (**a**), that the instrument has no uncontrolled common cause with the outcome (**b**, **c**), and that there should be no causal pathway from instrument to outcome, except via the exposure (**d**). In the present study, however, a causal effect of offspring BMI (instrument) on parental BMI (exposure) is implausible. Therefore, we must further assume that parental BMI is not causal of offspring BMI and that the common *genetic* and *environmental* factors causing the relation between offspring BMI and parental BMI are distinct from those confounding the association between BMI (exposure) and sick leave. In other words, for a valid instrument, there may be common causes of the instrument and exposure (which are independent of the outcome) and there may be common causes of the exposure and outcome (which are independent of the instrument), but there must be no (uncontrolled) common causes of the instrument and outcome. (*) Confounders e.g., education attainment, physical activity, smoking habits, alcohol consumption, occupation

The causal effect of BMI on sick leave was estimated using the instrumental variable ratio method [9, 24], with offspring BMI as an instrument. The numerator was the association between the instrument and the outcome, and the denominator was the association between the instrument and the exposure. The numerator was the natural logarithm of the HR per z-score of offspring BMI (from the Cox models). The denominator was the mean difference in z-score of parents BMI per SD of offspring BMI, obtained from linear regression. Adjustment sets were the same as described above for the Cox models in the corresponding numerator and denominator since it is plausible that these factors could influence offspring BMI (Fig. 2). These ratios were exponentiated to provide instrumental variable estimates of the HR per SD increase in z-score of BMI. We used Taylor series expansion to calculate the confidence intervals (CI) [25].

We constructed a bias component plot [26, 27] comparing the relative bias between conventional estimates and instrumental variable estimates, where the plotted components are proportional to the bias which would be a result of omitting the covariate. This involved making conventional (participants’ own BMI) and instrumental variable (offspring BMI) estimates of the effect of participants’ BMI on each covariate, using logistic or linear regression as appropriate with no adjustment. The estimates from the two different methods were scaled by the absolute magnitude of the larger of each component pair. In the plot, categorical covariates were dichotomized. The plotted components are only comparable between the methods in each covariate, and not between covariates or between men and women.

The proportional hazards assumption in Cox models was assessed by splitting the follow-up period at the median time to event and comparing the resulting HRs from the two periods using a postestimation command (i.e., testing if the effect of the exposure is different across time). Instrument strength was assessed using partial F-statistics and R-squared [28, 29]. To investigate if the instrumental variable estimates were influenced by the offspring potentially living at home, we performed the conventional and instrumental variable analyses using only participants with offspring ≥20 years old. To investigate the potential burden and thus increased risk of sick leave in participants having an offspring in one of the more extreme directions of BMI, we did the same analyses including only participants with offspring having a z-score of BMI between − 2 and 2. The precision of each estimated association was assessed by a 95% confidence interval. All analyses were performed using Stata statistical software (version 18) [30].

## Results

Mean age at participation was 45.3 (SD 8.2) years for women and 47.5 (SD 7.6) years for men, whereas their offspring’s mean age was 23.8 (SD 8.7) and 23.2 (SD 8.1) years, respectively. Mean BMI was 26.6 (SD 4.7) kg/m^2^ for women, 27.7 (SD 3.6) kg/m^2^ for men, 24.8 (SD 4.6) kg/m^2^ for offspring linked to women, and 24.7 (SD 4.6) kg/m^2^ for offspring linked to men. Smoking in participants was associated with higher BMI in their offspring, but lower own BMI (Table 1). Frequent alcohol consumption in participants was inversely associated with both own and offspring BMI, whereas low education, occupation EGP class ≥ III, and low physical activity were associated with higher own and offspring BMI.

**Table 1:** Participants’ characteristics stratified by sex and offspring body mass index (BMI)

Variable	Own z-score of BMI				Offspring z-score of BMI			
	<− 1	− 1 to 1	> 1	Per SD increase (95% CI)	<− 1	− 1 to 1	> 1	Per SD increase (95% CI)
*BMI of women and their offspring: women’s characteristics*								
Age (years)	44.9	45.4	44.7	− 0.00 (− 0.16 to 0.15)	46.5	45.0	45.7	− 1.35 (− 1.49 to − 1.21)
Z-score BMI	− 1.27	− 0.15	1.78		− 0.37	− 0.08	0.47	0.24 (0.22 to 0.25)
Low education^a^	50.7%	59.4%	69.2%	1.33 (1.27 to 1.38)	59.0%	57.7%	70.4%	1.06 (1.02 to 1.10)
Occupation EGP class ≥ III^b^	48.0%	54.4%	63.3%	1.25 (1.20 to 1.30)	53.9%	53.1%	64.4%	1.06 (1.03 to 1.10)
Current smoker^c^	27.8%	25.3%	23.4%	0.93 (0.89 to 0.97)	21.7%	24.2%	25.4%	1.14 (1.09 to 1.18)
High alcohol consumption^d^	17.7%	13.9%	5.2%	0.65 (0.60 to 0.69)	13.8%	13.6%	10.6%	0.87 (0.82 to 0.91)
Low physical activity^e^	36.1%	35.2%	45.9%	1.20 (1.15 to 1.24)	37.4%	35.7%	42.7%	1.06 (1.02 to 1.10)
*BMI of men and their offspring: men’s characteristics*								
Age (years)	47.6	47.6	47.5	0.21 (0.04 to 0.38)	48.7	47.3	48.0	− 1.16 (− 1.31 to − 1.01)
Z-score BMI	− 1.33	− 0.10	1.64		− 0.33	− 0.07	0.43	0.21 (0.19 to 0.23)
Low education^a^	66.8%	72.8%	81.4%	1.33 (1.26 to 1.40)	67.8%	72.1%	83.8%	1.14 (1.09 to 1.19)
Occupation EGP class ≥III^b^	57.7%	57.8%	64.5%	1.11 (1.06 to 1.16)	51.9%	57.7%	69.0%	1.16 (1.11 to 1.21)
Current smoker^c^	24.3%	19.9%	19.5%	0.93 (0.88 to 0.98)	19.4%	19.5%	25.6%	1.10 (1.05 to 1.15)
Alcohol consumption^d^	21.8%	21.7%	15.7%	0.85 (0.80 to 0.90)	21.5%	21.6%	17.6%	0.90 (0.86 to 0.95)
Low physical activity^e^	41.6%	42.4%	52.1%	1.20 (1.14 to 1.25)	41.3%	42.6%	50.5%	1.10 (1.05 to 1.14)

*BMI* Body mass index, *CI* Confidence interval, *EGP* the international Erikson Goldthorpe Porto-carero (EGP) social class scheme, *SD* Standard deviation

^a^Low education defined as lower than university level

^b^Lower class indicates higher socioeconomic status

^c^Daily and occasional smokers

^d^≥ 2–3 times a week

^e^Less than 3 h light and no hard activity

Continuous variables (age and BMI z-scores) are summarized as means in each category of BMI z-score and the association quantified as a mean difference. Binary variables are summarized as percentages in each category of BMI z-score and the association quantified as an odds ratio

### Offspring BMI as instrument

**Fig. 3:**
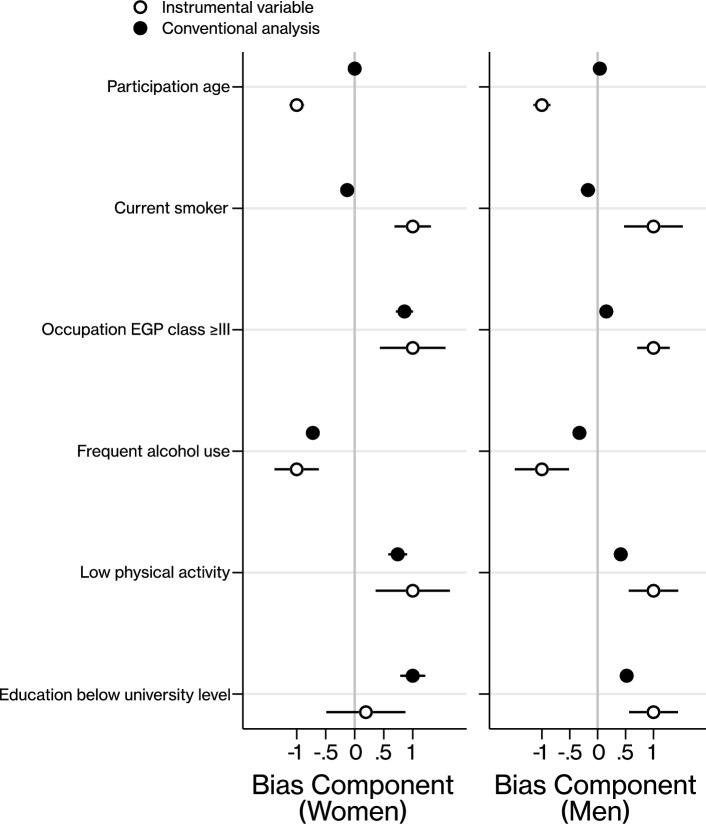
Bias components from measured covariates with corresponding 95% confidence intervals, plotted separately for women and men. The components are on a relative scale where each component pair is scaled by the absolute magnitude of the larger. Bias components are only comparable between the methods in each covariate, and not between covariates or between women and men

There was a strong association between offspring BMI and participants’ own BMI, with an adjusted mean difference in BMI z-score of 0.24 (95% CI 0.22–0.25) for women and 0.22 (95% CI 0.20–0.24) for men (Table 2). Corresponding partial R-squared and partial F-statistics were 5.73% and 607 for men and 6.17% and 855 for women. Analyses only adjusted for the interaction between date of birth and HUNT survey gave similar results (Table 2). The estimates and the corresponding R-squared and F-statistic were also similar when only using offspring ≥ 20 years old as instruments (Table S1), and when only using offspring in the range of z-score between − 2 and 2 (Table S2). Bias components for measured covariates were greater in the instrumental variable analysis compared to conventional analysis except for education in women (Fig. 3). However, there was considerable overlap in CI of the bias components among women, except participation age and current smoker. This suggests that our IV approach will have at least the same amount of residual confounding in relative terms, although with more uncertainty amongst women.

**Table 2:** Strength of instruments presented as mean difference in z-score of body mass index (BMI) per standard deviation of offspring z-score of BMI, partial R-squared and partial F-statistic

Pair	Model	N	MD in z-score of BMI (95% CI)^c^	R^2^, %^d^	F^e^
Women-offspring	Unadjusted^a^	12,312	0.24 (0.23–0.26)	6.71	886
Women-offspring	Adjusted^b^	12,312	0.24 (0.22–0.25)	6.17	855
Men-offspring	Unadjusted^a^	9606	0.22 (0.21–0.24)	6.14	629
Men-offspring	Adjusted^b^	9606	0.22 (0.20–0.24)	5.73	607

*BMI* Body mass index, *MD* Mean difference, *N* number of observations

^a^Adjusted for date of birth*HUNT wave

^b^Adjusted for date of birth*HUNT wave, education attainment, occupation class, smoking status, alcohol consumption, and physical activity

^c^Per standard deviation increase in offspring z-score of BMI

^d^Partial R-squared which reflect the proportion of BMI that is explained by offspring BMI (instrument)

^e^Partial F-statistics which measure instrument strength

### BMI and risk of long-term sick leave

**Fig. 4:**
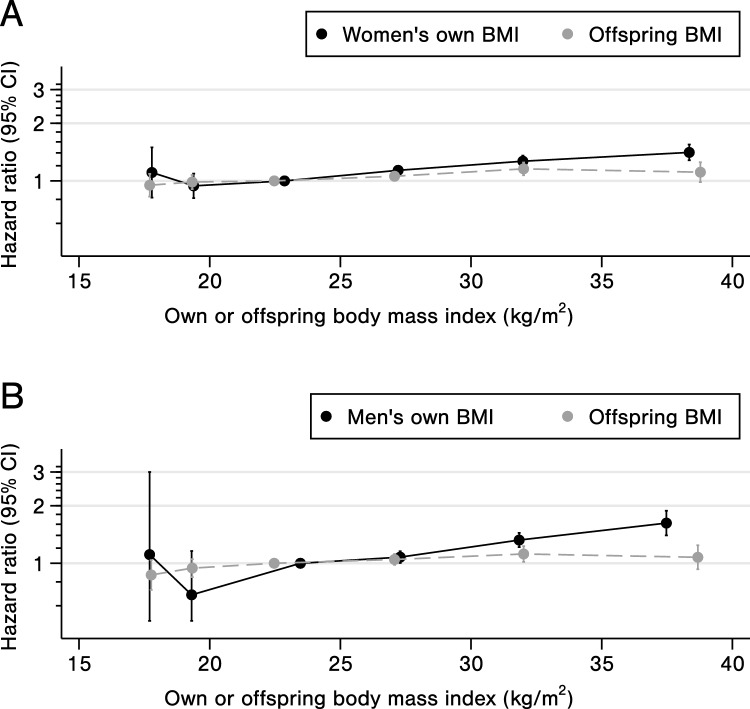
**A** Women **B** Men. Associations between all-cause sick leave and parents own body mass index (BMI) or offspring BMI. Results from Cox models adjusted for interaction term between age and HUNT survey, education, smoking status, alcohol consumption and physical activity. BMI was categorized as < 18.5, 18.5–19.9, 20–24.9, 25–29.9, 30–34.9 and ≥ 35 kg/m^2^. Hazard ratios with corresponding 95% confidence interval are plotted as category means relative to a BMI reference level of 20–24.9 kg/m^2^

The incidence rates for all-cause sick leave were 115 per 1000 person years for women with a median follow up time of 4.1 years (mean, 5.8 years), and 78 per 1000 person years for men with a median follow up time of 5.4 years (mean, 6.9 years). The corresponding rates for sick leave due to musculoskeletal disorders were 50 and 37, whereas for mental health disorders they were 22 and 10, respectively. There was a J-shaped association between BMI and risk of all-cause sick leave, while the association was more linear when using offspring BMI as instrument (Fig. 4).

Conventional analyses suggest an increased risk of long-term sick leave in both women and men with higher levels of BMI (Table 3). Adjusted HRs per SD increase in z-score of BMI ranged from 1.04 (95% CI 0.99–1.08) for mental health disorders in women to 1.17 (95% CI 1.13–1.22) for musculoskeletal disorders in men. The instrumental variable approach supports that higher BMI increases the risk of long-term sick leave, although some estimates were imprecise. In particular, the estimate for sick leave due to mental health disorders in men was close to the null, but quite strong associations in either direction could not be ruled out (Table 3).

**Table 3:** Hazard ratios (HR) for risk of sick leave by z-scores of offspring body mass index (BMI), own BMI, and offspring BMI as an instrumental variable (IV) for own BMI

	Unadjusted^a^ HR (95% CI) per SD increase in			Adjusted^b^ HR (95% CI) per SD increase in		
	Offspring BMI	Own BMI	Own BMI (IV)	Offspring BMI	Own BMI	Own BMI (IV)
*Sick leave in women*						
All-cause	1.07 (1.04–1.09)	1.11 (1.09–1.14)	1.31 (1.19–1.43)	1.05 (1.03–1.08)	1.10 (1.08–1.13)	1.24 (1.13–1.36)
Musculoskeletal disorders	1.07 (1.04–1.10)	1.15 (1.12–1.18)	1.30 (1.15–1.46)	1.04 (1.01–1.07)	1.12 (1.09–1.15)	1.17 (1.04–1.32)
Mental health disorders	1.04 (1.00–1.08)	1.02 (0.98–1.06)	1.16 (0.99–1.36)	1.04 (1.00–1.08)	1.04 (0.99–1.08)	1.17 (0.99–1.39)
*Sick leave in men*						
All-cause	1.05 (1.02–1.08)	1.17 (1.13–1.20)	1.24 (1.10–1.41)	1.02 (0.99–1.05)	1.15 (1.11–1.18)	1.11 (0.97–1.26)
Musculoskeletal disorders	1.06 (1.02–1.10)	1.19 (1.15–1.24)	1.29 (1.10–1.52)	1.02 (0.99–1.06)	1.17 (1.13–1.22)	1.11 (0.94–1.31)
Mental health disorders	0.99 (0.93–1.06)	1.08 (1.00–1.15)	0.96 (0.72–1.27)	1.00 (0.94–1.06)	1.08 (1.00–1.15)	0.99 (0.74–1.33)

*BMI* Body mass index, *CI* Confidence interval, *HR* Hazard ratio, *IV* Instrumental variable, *SD* Standard deviation

^a^Adjusted for date of birth*HUNT wave

^b^Adjusted for date of birth*HUNT wave, education attainment, occupation class, smoking status, alcohol consumption, and physical activity

Sensitivity analyses including only participants with offspring ≥ 20 years old provided similar estimates in both conventional and instrumental variable analyses for women and men, although instrumental variable estimates for sick leave due to all cause and musculoskeletal disorders in men suggest a further increased risk compared to the main analyses (Table S3). When we included only participants who had offspring with a z-score between -2 and 2, the estimates remained similar to those of the main analyses, except for mental health disorders in men where the instrumental variable estimates weakly suggested a reduced risk of sick leave with increasing BMI (Table S4). There was no evidence of violation of the proportional hazards assumption when comparing HRs from the early and late follow-up periods (i.e., splitting follow-up time at the median value) (Table S5).

## Discussion

Our conventional analyses showed an increased risk of long-term sick leave with higher BMI, although with a slightly J-shaped association for all-cause sick leave. When using offspring BMI as an instrument, the effect was more linear, which suggests that the risk could be overestimated at the lower end of the BMI scale when using conventional analyses. This indicates that the influence of underlying disease is attenuated by the instrumental variable analysis that is likely to reduce the role of reverse causation. Overall, the instrumental variable analysis supported a causal relationship where higher BMI was associated with an increased risk of sick leave. This was observed for all-cause sick leave and sick leave due to musculoskeletal disorders among both men and women. An increased risk of sick leave due to mental health disorders was observed only for women.

A previous study using offspring body weight as an instrument for mothers body weight did not find an effect on employment disability [10]. The authors concluded that the observed correlation between higher weight and employment disability could be due to reverse causation (i.e., disability causing weight gain) or unmeasured confounders. In our study, the association between BMI and offspring BMI showed good instrument strength, indicating that our results were less likely to be prone to weak instrument bias. However, for long-term sick leave, common causes between BMI and offspring BMI might also affect the risk of sick leave. Socioeconomic and educational attainment are associated with both BMI in offspring [31, 32] and sick leave [1], although with conflicting findings [33]. When adding educational attainment and occupational class in the fully adjusted analysis in our study, the instrumental variable estimates of men were slightly more attenuated than the corresponding estimates of women. Although our instrumental variable estimates may be vulnerable to confounding of the instrument-outcome relationship (Fig. 2), the instrumental variable estimates are more robust to bias due to reverse causation since the instrument is unlikely to be directly influenced by the outcome (Fig. 2). The method can therefore provide important triangulation of evidence, as the sources of bias in instrumental variable analysis will differ from those in conventional analysis.

An assumption for the instrumental variable analysis is that offspring BMI should not directly affect risk of sick leave in their parents. Offspring with a BMI on either extreme of the scale might be prone to other conditions that can affect their parents’ mental and physical health, and thus their risk of long-term sick leave. In a sensitivity analysis where we excluded participants with offspring who had a z-score of <-2 or >2 the estimated effect of BMI on sick leave did not change considerably, except for sick leave due to mental health disorders in men which moved in an inverse direction.

## Strengths and limitations

The strengths of this study include the large study population, triangulation of methods and the intergenerational, prospective design with long follow-up using nationwide registries. However, some limitations should be considered. Firstly, offspring BMI have previously been used primarily as a valid instrument for parents own BMI to overcome bias due to reverse causation by ill health. However, when used to estimate risk of sick leave, its plausible that our estimates suffer from residual confounding due to behavioral and/or socioeconomic factors. Generally, instrumental variable approaches typically depend on strong assumptions, necessitating careful interpretation of the results. It is also important to note that since this study includes family trios the results for women and men are not independent of each other, i.e., there is overlapping information on offspring BMI. Secondly, although BMI is considered a good measure for health on a population level, differences in body composition on an individual level may mislead interpretation of the estimated risk of sick leave. While weight and height (BMI) were measured by professionals, some of the adjustment variables were assessed using self-reported questionnaires, and we can therefore not rule out bias due to misclassification or measurement error. Furthermore, since instrumental variable approaches require large sample sizes, we have limited statistical power to stratify by offspring age and sex. Finally, we cannot rule out bias from the selection of participants as both parents and offspring had to participate in the surveys. However, the direction of this bias is unclear. Selection bias due to missing data on covariates is unlikely since less than 3% had missing data on covariates. Finally, we cannot rule out the possibility that BMI is linked to competing events occurring before a sick leave episode, which may lead to an underestimation of the effect of BMI on sick leave risk.

## Conclusion

An instrumental variable approach to investigate BMI as a risk factor for long-term sick leave supports a possible causal role of higher BMI in increasing the risk of long-term sick leave due to all-cause and musculoskeletal disorders. For mental health disorders the increased risk was only observed for women, although results should be interpreted cautiously given possible remaining bias in the instrumental variable estimates. Although offspring BMI seems a suitable proxy for own BMI, it is not independent of shared confounding when examining risk of long-term sick leave. The instrumental variable method should be considered complementary to conventional regression analysis since potential biasing paths will be different, and thus contribute to triangulation of evidence.

## Supplementary Information

Below is the link to the electronic supplementary material.

Supplementary file1 (DOCX 38 kb)

## Data Availability

The data underlying this article were provided by HUNT (https://www.ntnu.edu/hunt/), NAV (https://www.nav.no/) and Statistics Norway (https://www.ssb.no/en) under license, and so are not publicly available. Enquiries about the data materials used in this study can be sent by mail to lene.aasdahl@ntnu.no. The Stata code used to replicate the analysis and visualization used in the paper is openly available on GitHub at https://github.com/karolmoe/IV-BMI-sickleave.
