# Supplementary material for: An instrumental variable analysis of body mass index and risk of long-term sick leave: the HUNT Study, Norway
Source: Eur J Epidemiol. 2025 Sep 4;40(10):1221–30. doi: 10.1007/s10654-025-01299-6 (PMC12660429; doi:10.1007/s10654-025-01299-6)
Supplement: Supplementary file 1 — Supplementary file1 (DOCX 38 kb) [file 10654_2025_1299_MOESM1_ESM.docx]

**An instrumental variable analysis of body mass index and risk of long-term sick leave: the HUNT Study, Norway**

Karoline Moe(0009-0007-2758-6688)^1^, Eivind Schjelderup Skarpsno(0000-0002-4135-0408)^1,2^, Tom Ivar Lund Nilsen(0000-0001-8251-3544)^1,3^, Silje L. Kaspersen(0000-0001-8343-0871)^1,4^, Solveig Osborg Ose(0000-0001-5361-7571)^4^, David Carslake(0000-0003-2916-4546)^5,6^, Paul Jarle Mork(0000-0003-3355-2680)^1^, Lene Aasdahl(0000-0003-4276-1345)^1,7^

1) Department of Public Health and Nursing, Faculty of Medicine and Health Sciences, Norwegian University of Science and Technology, Trondheim, Norway
2) Department of Neurology and Clinical Neurophysiology, St. Olavs Hospital, Trondheim, Norway
3) Clinic of Emergency Medicine and Prehospital Care, St. Olav's Hospital, Trondheim University Hospital, Trondheim, Norway

4) Department of Health Research, SINTEF Digital, Trondheim, Norway
5) Medical Research Council (MRC) Integrative Epidemiology Unit at the University of Bristol, Bristol, UK
6) Population Health Sciences, Bristol Medical School, Faculty of Health Sciences, University of Bristol, Bristol, UK
7) Unicare Helsefort Rehabilitation Centre, Rissa, Norway

**Corresponding author:**

Karoline Moe

Department of Public Health and Nursing, Norwegian University of Science and Technology (NTNU). Email: karoline.moe@ntnu.no

**Contact address**

Department of Public Health and Nursing, Norwegian University of Science and Technology (NTNU), Postboks 8905, 7491 Trondheim, Norway.

**Other variables**

Smoking status consisted originally of four categories in HUNT3 (“Never smoked”, “ex-smoker”, “occasional smoker”, and “daily smoker”), and five categories in HUNT 4 (“never smoked”, “ex-occasional smoker”, “ex-daily smoker”, “current occasional smoker”, and “current daily smoker”). The response options “ex-daily smoker” and “ex-occasional smoker” in HUNT4 were collapsed into “ex-smoker” to harmonize with HUNT3. Alcohol consumption in HUNT4 was assessed by the question “About how often in the last 12 months did you drink alcohol? (Do not include low-alcohol beer)” with six response options: “never drunk alcohol”, “not at all the last year”, “once a month or less”, “2-4 times a month”, “2-3 times a week”, and “4 times or more a week”. HUNT3 had two additional response options which were collapsed into “once a month or less”, and “2-4 times a month” to harmonize with HUNT4. In the HUNT surveys, EGP class I represents self-employed higher grade professionals (e.g. dentist, lawyer), and management positions in public or private organizations, class II is professional occupations (e.g. nurse, technician, teacher), class III represents non-professional occupations (e.g. shop, office, public service), class IV is other self-employed, farmers, foresters, and fishermen, class V+VI is skilled manual workers, artisans, and supervisors of manual workers, and finally, class VII is unskilled manual workers and drivers (1).

**Table S1.** Strength of instruments presented as mean difference in z-score of body mass index (BMI) per standard deviation of offspring z-score of BMI, partial R-squared and partial F-statistic. Restricted sample of participants with offspring ≥20 years.

| Pair | Model | N | MD in z-score of BMI (95% CI)^c^ | R-squared^d^ | F^e^ |
| --- | --- | --- | --- | --- | --- |
| Women-offspring | Unadjusted^a^ | 7,021 | 0.26 (0.23-0.28) | 6.65 | 501 |
| Women-offspring | Adjusted^b^ | 7,021 | 0.25 (0.23-0.27) | 6.06 | 481 |
| Men-offspring | Unadjusted^a^ | 5,418 | 0.26 (0.24-0.28) | 7.34 | 430 |
| Men-offspring | Adjusted^b^ | 5,418 | 0.25 (0.23-0.28) | 6.75 | 407 |

Abbreviations: BMI = body mass index, MD = mean difference, N = number of observations. ^a^ Adjusted for date of birth*HUNT wave.
^b^ Adjusted for date of birth*HUNT wave, education attainment, occupation class, smoking status, alcohol consumption, and physical activity.
^c^ Per standard deviation increase in offspring z-score of BMI. ^d^ Partial R-squared which reflect the proportion of BMI that is explained by offspring BMI (instrument).
^e^ Partial F-statistics which measure instrument strength.

**Table S2.** Strength of instruments presented as mean difference in z-score of body mass index (BMI) per standard deviation of offspring z-score of BMI, partial R-squared and partial F-statistic. Restricted sample of participants with z-score of offspring BMI between -2 to 2.

| Pair | Model | N | MD in z-score of BMI (95% CI)^c^ | R-squared^d^ | F^e^ |
| --- | --- | --- | --- | --- | --- |
| Women-offspring | Unadjusted^a^ | 12,032 | 0.24 (0.22-0.26) | 5.48 | 698 |
| Women-offspring | Adjusted^b^ | 12,032 | 0.23 (0.22-0.25) | 5.18 | 692 |
| Men-offspring | Unadjusted^a^ | 9,388 | 0.22 (0.20-0.24) | 4.97 | 491 |
| Men-offspring | Adjusted^b^ | 9,388 | 0.22 (0.20-0.23) | 4.71 | 481 |

Abbreviations: BMI = body mass index, MD = mean difference, N = number of observations. ^a^ Adjusted for date of birth*HUNT wave.
^b^ Adjusted for date of birth*HUNT wave, education attainment, occupation class, smoking status, alcohol consumption, and physical activity.
^c^ Per standard deviation increase in offspring z-score of BMI. ^d^ Partial R-squared which reflect the proportion of BMI that is explained by offspring BMI (instrument).
^e^ Partial F-statistics which measure instrument strength.

|  | Only offspring ≥20 years | |  | Original study sample | |
| --- | --- | --- | --- | --- | --- |
|  | Adjusted^a^ HR (95% CI)  per SD increase in | |  | Adjusted^a^ HR (95% CI)  per SD increase in | |
| Sick leave in women | Own BMI | Own BMI (IV) |  | Own BMI | Own BMI (IV) |
| All-cause | 1.11 (1.08-1.15) | 1.29 (1.14-1.46) |  | 1.10 (1.08-1.13) | 1.24 (1.13-1.36) |
| Musculoskeletal disorders | 1.14 (1.10-1.18) | 1.21 (1.04-1.41) |  | 1.12 (1.09-1.15) | 1.17 (1.04-1.32) |
| Mental health disorders | 1.02 (0.96-1.08) | 1.16 (0.92-1.48) |  | 1.04 (0.99-1.08) | 1.17 (0.99-1.39) |
| Sick leave in men | | | | | |
| All-cause | 1.15 (1.11-1.20) | 1.21 (1.04-1.40) |  | 1.15 (1.11-1.18) | 1.11 (0.97-1.26) |
| Musculoskeletal disorders | 1.19 (1.13-1.25) | 1.20 (1.00-1.45) |  | 1.17 (1.13-1.22) | 1.11 (0.94-1.31) |
| Mental health disorders | 1.06 (0.97-1.17) | 0.98 (0.68-1.43) |  | 1.08 (1.00-1.15) | 0.99 (0.74-1.33) |

**Table S3**. Hazard ratios (HR) for risk of sick leave by own body mass index (BMI), and offspring BMI as an instrumental variable (IV) for own BMI. Restricted sample of participants with offspring ≥20 years compared with the adjusted main results (Table 3).

Abbreviations: BMI = body mass index, CI = confidence interval, HR = hazard ratio, IV = instrumental variable, SD = standard deviation.
^a^ Adjusted for date of birth*HUNT wave, education attainment, occupation class, smoking status, alcohol consumption, and physical activity.

**Table S4.** Hazard ratios (HR) for risk of sick leave by own body mass index (BMI), and offspring BMI as an instrumental variable (IV) for own BMI. Restricted sample of participants with z-score of offspring BMI between -2 to 2 compared with the adjusted main results (Table 3).

|  | Offspring z-score range -2 to 2 | |  | Original study sample | |
| --- | --- | --- | --- | --- | --- |
|  | Adjusted^a^ HR (95% CI)  per SD increase in | |  | Adjusted^a^ HR (95% CI)  per SD increase in | |
| Sick leave in women | Own BMI | Own BMI (IV) |  | Own BMI | Own BMI (IV) |
| All-cause | 1.10 (1.08-1.13) | 1.22 (1.10-1.36) |  | 1.10 (1.08-1.13) | 1.24 (1.13-1.36) |
| Musculoskeletal disorders | 1.12 (1.09-1.16) | 1.13 (0.99-1.29) |  | 1.12 (1.09-1.15) | 1.17 (1.04-1.32) |
| Mental health disorders | 1.03 (0.99-1.08) | 1.13 (0.94-1.37) |  | 1.04 (0.99-1.08) | 1.17 (0.99-1.39) |
| Sick leave in men | | | | | |
| All-cause | 1.14 (1.11-1.18) | 1.09 (0.94-1.26) |  | 1.15 (1.11-1.18) | 1.11 (0.97-1.26) |
| Musculoskeletal disorders | 1.16 (1.12-1.21) | 1.11 (0.93-1.34) |  | 1.17 (1.13-1.22) | 1.11 (0.94-1.31) |
| Mental health disorders | 1.06 (0.98-1.14) | 0.83 (0.60-1.16) |  | 1.08 (1.00-1.15) | 0.99 (0.74-1.33) |

Abbreviations: BMI = body mass index, CI = confidence interval, HR = hazard ratio, IV = instrumental variable, SD = standard deviation.
^a^ Adjusted for date of birth*HUNT wave, education attainment, occupation class, smoking status, alcohol consumption, and physical activity.

**Table S5**. The proportional hazards assumption in Cox models was assessed by splitting follow-up at median time to event and compare the resulting Hazard ratios (HRs) from the two periods.

| **Who** | **Outcome** | **Exposure** | **Median age for outcome** | **Early HR** | **Early 95% CI** | **Late HR** | **Late 95% CI** | **Early (log)HR = Late (log)HR, p-value** |
| --- | --- | --- | --- | --- | --- | --- | --- | --- |
| Women | All-cause | Own BMI | 49.5 | 1.10 | 1.06-1.13 | 1.11 | 1.08-1.15 | 0.60 |
| Men | All-cause | Own BMI | 52.9 | 1.14 | 1.09-1.19 | 1.15 | 1.11-1.20 | 0.76 |
| Women | Musculoskeletal | Own BMI | 50.6 | 1.12 | 1.07-1.16 | 1.13 | 1.09-1.18 | 0.63 |
| Men | Musculoskeletal | Own BMI | 53.2 | 1.17 | 1.10-1.23 | 1.18 | 1.12-1.24 | 0.83 |
| Women | Mental | Own BMI | 48.7 | 1.04 | 0.98-1.11 | 1.03 | 0.97-1.09 | 0.75 |
| Men | Mental | Own BMI | 51.1 | 1.08 | 0.98-1.19 | 1.07 | 0.97-1.18 | 0.92 |
| Women | All-cause | Offspring BMI | 49.5 | 1.03 | 1.00-1.07 | 1.06 | 1.03-1.10 | 0.21 |
| Men | All-cause | Offspring BMI | 52.9 | 1.01 | 0.97-1.05 | 1.04 | 1.00-1.08 | 0.38 |
| Women | Musculoskeletal | Offspring BMI | 50.6 | 1.02 | 0.98-1.06 | 1.05 | 1.01-1.09 | 0.40 |
| Men | Musculoskeletal | Offspring BMI | 53.2 | 1.00 | 0.95-1.05 | 1.04 | 0.99-1.10 | 0.26 |
| Women | Mental | Offspring BMI | 48.7 | 1.04 | 0.99-1.10 | 1.02 | 0.97-1.09 | 0.65 |
| Men | Mental | Offspring BMI | 51.1 | 0.96 | 0.88-1.05 | 1.03 | 0.94-1.13 | 0.28 |

Abbreviations: CI- confidence interval, HR- hazard ratio

**Table S6**. Age and sex specific z-score of body mass index (BMI) in correspondence to kilograms (kg), presented for women, men, and their offspring.

| Women |  |  |  |  |
| --- | --- | --- | --- | --- |
| Age (years) | 20-29.9 | 30-39.9 | 40-49.9 | 50-60 |
| Mean height (m) | 1.66 | 1.67 | 1,67 | 1.65 |
| Mean BMI (kg/m^2^) | 25.3 | 26.3 | 26.6 | 27.1 |
| SD of BMI | 4.69 | 5.00 | 4.69 | 4.40 |
| Z-score | 1 | 1 | 1 | 1 |
| Z-score into kilograms^a^ | 12.9 | 14.0 | 13.1 | 12.0 |
| Offspring of the women |  |  |  |  |
| Mean height (m) | 1.70 | 1.73 | 1,73 | 1.73 |
| Mean BMI (kg/m^2^) | 23.5 | 23.8 | 24.5 | 25.9 |
| SD of BMI | 4.37 | 4.34 | 4.46 | 4.85 |
| Z-score | 1 | 1 | 1 | 1 |
| Z-score into kilograms^a^ | 12.6 | 13.0 | 13.4 | 14.5 |
| Men |  |  |  |  |
| Age (years) | 20-29.9 | 30-39.9 | 40-49.9 | 50-60 |
| Mean height (m) | 1.81 | 1.80 | 1,80 | 1.79 |
| Mean BMI (kg/m^2^) | 26.18 | 27.3 | 27.8 | 27.9 |
| SD of BMI | 4.16 | 3.60 | 3.60 | 3.58 |
| Z-score | 1 | 1 | 1 | 1 |
| Z-score into kilograms^a^ | 13.6 | 11.7 | 11.7 | 11.5 |
| Offspring of the men |  |  |  |  |
| Mean height (m) | 1.70 | 1.73 | 1,73 | 1.73 |
| Mean BMI (kg/m^2^) | 22.9 | 23.6 | 24.3 | 25.5 |
| SD of BMI | 3.77 | 4.23 | 4.44 | 4.82 |
| Z-score | 1 | 1 | 1 | 1 |
| Z-score into kilograms^a^ | 10.9 | 12.7 | 13.3 | 14.4 |

^a^ Conversion of one z-score unit of BMI to additional mass in kg for a person of average height in this group; SD of BMI * mean height^2^
Abbreviations: BMI = body mass index, kg = kilograms, m = meters. SD = standard deviation.
Per SD increase in z-score of BMI for a woman aged between 30-39.9 years corresponds to 14.0 kilograms. Similarly, when using offspring BMI as instrument, per SD increase of z-score of offspring BMI for a woman between 30-39.9 years corresponds to 13.0 kilograms.

**References**

1. Krokstad S, Ringdal K, Westin S. Classifying people by social class in population based health surveys. Norsk epidemiologi. 2002;12(1):19-25.
